# *Streptomyces smaragdinus* sp. nov., isolated from the gut of the fungus growing-termite *Macrotermes natalensis*

**DOI:** 10.1099/ijsem.0.004478

**Published:** 2020-09-24

**Authors:** Jan W. Schwitalla, René Benndorf, Karin Martin, John Vollmers, Anne-Kristin Kaster, Z. Wilhelm de Beer, Michael Poulsen, Christine Beemelmanns

**Affiliations:** ^1^​ Leibniz Institute for Natural Product Research and Infection Biology e. V., Hans-Knöll-Institute, Beutenbergstraße 11a, 07745 Jena, Germany; ^2^​ Institute for Biological Interfaces (IBG 5), Karlsruhe Institute of Technology, Hermann-von-Helmholtz-Platz 1, 76344 Eggenstein-Leopoldshafen, Germany; ^3^​ Department of Microbiology and Plant Pathology, Forestry and Agriculture Biotechnology Institute, University of Pretoria, 0028 Hatfield, South Africa; ^4^​ University of Copenhagen, Department of Biology, Section for Ecology and Evolution, Universitetsparken 15, 2100 Copenhagen East, Denmark

**Keywords:** *Macrotermes natalensis*, *Streptomyces*, termite gut

## Abstract

The taxonomic position of a novel aerobic, Gram-positive actinobacteria, designated strain RB5^T^, was determined using a polyphasic approach. The strain, isolated from the gut of the fungus-farming termite *Macrotermes natalensis*, showed morphological, physiological and chemotaxonomic properties typical of the genus *Streptomyces *. Based on 16S rRNA gene sequence analysis, the closest phylogenetic neighbour of RB5^T^ was *Streptomyces polyrhachis* DSM 42102^T^ (98.87 %). DNA–DNA hybridization experiments between strain RB5^T^ and *S. polyrhachis* DSM 42102^T^ resulted in a value of 27.4 % (26.8 %). The cell wall of strain RB5^T^ contained ll-diaminopimelic acid as the diagnostic amino acid. Mycolic acids and diagnostic sugars in whole-cell hydrolysates were not detected. The strain produced the following major phospholipids: diphosphatidylglycerol, phosphatidylethanolamine, phosphatidylinositol, phosphatidylinositol-mannoside and phosphatidylserine. The menaquinone profile showed hexa- and octahydrogenated menaquinones containing nine isoprene units [MK-9(H_6_) and MK-9(H_8_)]. The strain exhibited a fatty acid profile containing the following major fatty acids: 12-methyltridecanoic acid (iso-C_14 : 0_) 12-methyltetradecanoic acid (anteiso-C_15 : 0_), 13-methyltetradecanoic acid (iso-C_15 : 0_) and 14-methylpentadecanoic acid (iso-C_16 : 0_). Here, we propose a novel species of the genus *Streptomyces – Streptomyces smaragdinus* with the type strain RB5^T^ (=VKM Ac-2839^T^=NRRL B65539^T^).

## Introduction

The class *Actinobacteria* harbours numerous genera including the well-known genus *Streptomyces *, which was first introduced by Waksman and Henrici [1]. *Streptomyces* species are characterized as aerobic, Gram-positive, filamentous bacteria, which form branched substrate mycelium and aerial hyphae. Members of the genus have a high G+C content and contain the diagnostic ll-diaminopimelic acid in the cell-wall peptidoglycan [2, 3]. In addition to being reported as typical soil bacteria, members of the genus *Streptomyces* have also been found to act as protective symbionts for different insect species [4–6]. Research efforts into the chemical ecology of *Streptomyces* have also been stimulated by the fact that the genus is known for its enormous biosynthetic and metabolic capability to produce small bioactive molecules [7, 8]. In this study, we focused on the isolation and characterization of culturable microbes from fungus-growing termites of the genus *Macrotermes natalensis*. Preceding microbiome studies indicated a highly diverse, but stable microbiome within the termite gut and fungus comb of the termite nest [9, 10]. In particular, comb analysis revealed a strong abundance of members of the phyla *Firmicutes *, *Bacteroidetes *, *Proteobacteria* and *Actinobacteria *, with *Streptomyces* as one of the most dominant genera of the latter. Here, we speculated that the specialized symbiotic ecosystem also represents an unexplored source of new bacterial species that assist in the breakdown of organic material to sustain growth of the termite colony. Using a cultivation-based approach, we isolated and characterized a novel strain, RB5^T^, comprising a new species of the genus *Streptomyces *, hereafter named as *Streptomyces smaragdinus*.

## Isolation and ecology

In February 2015 symbiotic actinomycetes associated with fungus-growing termites of the genus *Macrotermes natalensis* were collected from colony Mn160 (25° 44′ 34.7″ S 28° 15′ 38.7″ E, Pretoria, South Africa) and strain RB5^T^ was isolated from the guts of termite workers [11]. Colonies growing on chitin agar supplemented with 0.05 g l^−1^ cycloheximide were transferred onto yeast extract–malt extract agar (ISP2). Isolated pure strain RB5^T^ was maintained on ISP2 at 30 °C and as glycerol suspensions (25 %, v/v) at −80 °C.

## Phylogeny

Extraction of genomic DNA and PCR amplification of the 16S rRNA gene of RB5^T^ was performed as described previously [11]. Furthermore, the sequence for the 16S rRNA gene of RB5^T^ was extracted from whole genome data using Artemis [12] and the near-complete 16S rRNA gene sequence was used for phylogenetic analyses. blastn analysis was performed using the NCBI database. The results indicated that strain RB5^T^ is a member of the genus *Streptomyces *. The 16S rRNA gene sequences of published *Streptomyces* reference strains were downloaded from the NCBI ‘refseq’ database [13] and pairwise sequence similarities were calculated using the method recommended by Meier-Kolthoff *et al.* [14] on the GGDC web server [15]. sina sequence alignment was used for 16S rRNA gene sequence alignments [16]. Phylogenetic trees were reconstructed with mega software (version 7.0.26) [17] using the neighbour-joining [18] and maximum-likelihood algorithms [19]. The evolutionary distance model of Tamura [20] was used to generate evolutionary distance matrices for the maximum-likelihood and neighbour-joining algorithms with deletion of complete gaps and missing data. For the maximum-likelihood algorithm, discrete gamma distribution was used (*+G*) and the rate variation model allowed for some sites to be evolutionarily invariable (*+I*). For the neighbour-joining algorithm, rate variation among sites was modelled with a gamma distribution. The confidence values of nodes were evaluated by bootstrap analysis based on 1000 re-samplings [21].

Strain RB5^T^ showed highest 16S rRNA similarity to *Streptomyces polyrhachis* DSM 42102^T^ (=NEAU-ycm1^T^; 98.87 %). Lower levels of 16S rRNA gene sequence similarity (<98.70 %) were found to all other *Streptomyces* type strains (Table S1, available in the online version of this article).

**Fig. 1.**
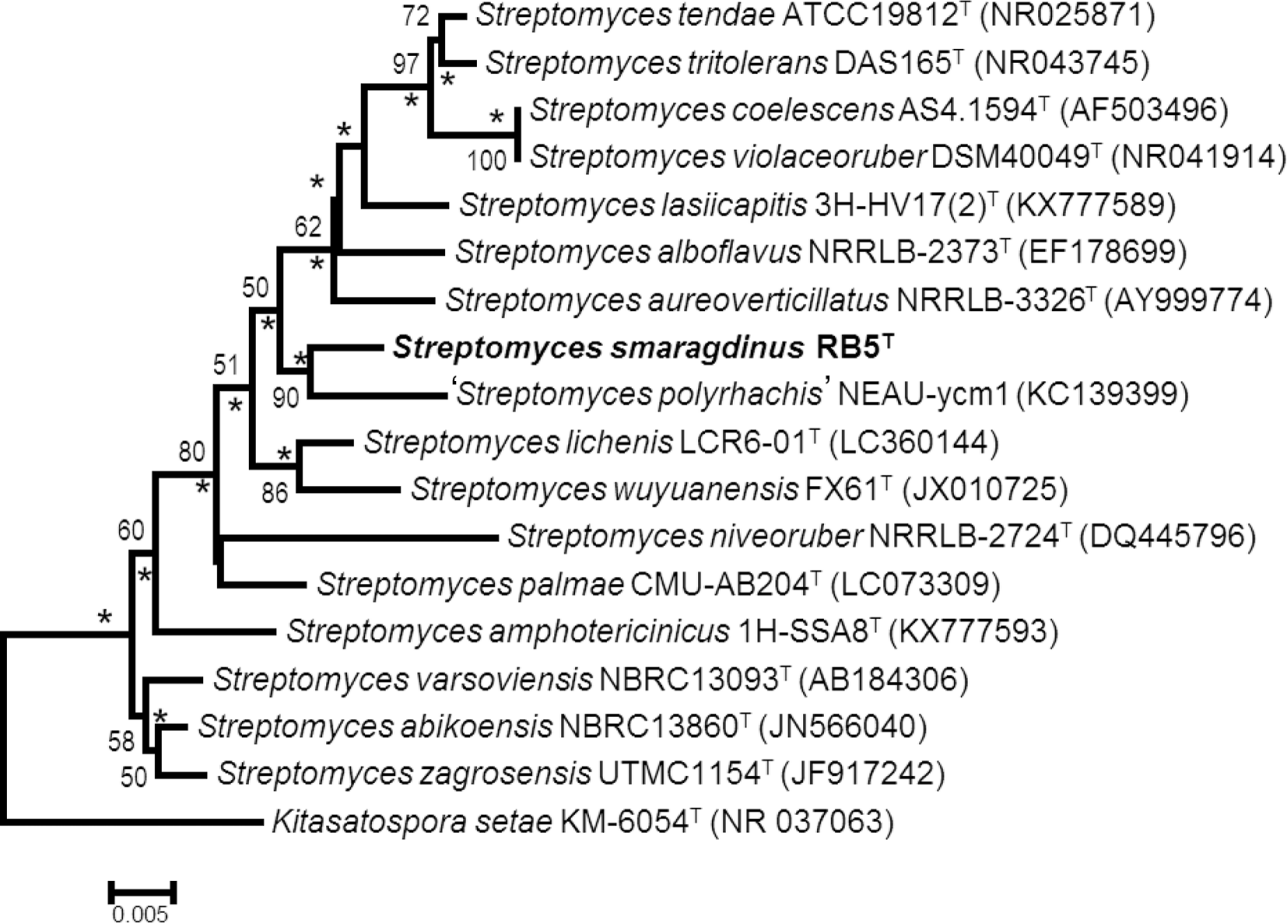
Neighbour-joining tree based on almost-complete 16S rRNA gene sequences showing relationships between strain RB5^T^ and species of the genus *Streptomyces. Kitasatospora setae* KM-6054^T^ was used as an outgroup. Asterisks donate branches that were also recovered in the maximum-likelihood tree. Only bootstrap values above 50 % (percentages of 1000 pseudo-replications) are shown. Bar, 0.005 substitutions per nucleotide position.

Phylogenetic analysis based on the 16S rRNA gene showed that strain RB5^T^ formed a distinct branch with *S. polyrhachis* DSM42101^T^ in the neighbour-joining tree (Fig. 1) and the topology structure was also supported by the maximum-likelihood tree (Fig. S1). These findings are supported by high bootstrap values of 90 % in both trees.

## Genome features

Genome sequencing of RB5^T^ was performed as previously described and the G+C content of the DNA was determined from whole sequence data [11]. DNA–DNA hybridization (DDH) was performed by the German Collection of Microorganisms and Cell Cultures GmbH (DSMZ) Identification service as a classical wet-lab experiment. The required DNA was obtained as described by Cashion *et al.* [22] and DDH experiments were performed in duplicate according to the methods of De Ley *et al.* [23] and Huss *et al.* [24].

The wet-lab DDH value of RB5^T^ and the closest relative *S. polyrhachis* DSM 42102^T^ was 27.4 % (26.8 %). This value is below the threshold value of 70 % for the delineation of bacterial species recommended by Meier-Kolthoff *et al.* [14] and Wayne *et al.* [25].

The genome of RB5^T^ was 7.9 Mb in size (107 contigs with an N50 value of 161 565 bp and an L50 value of 13, total CDS were 7136 and the genome had a completeness of 100 % (Table S2). The G+C content of the type strain was determined as 72 mol%, which is typical for the genus of *Streptomyces* [26, 27].

## Physiology and chemotaxonomy

Morphological characteristics of the strains were determined on cultures grown for 5–14 days on ISP2 agar at 30 °C using a light microscope (Imager M2, Carl Zeiss GmbH) and a field emission scanning electron microscope. Scanning electron microscopy was performed as described by Groth *et al.* [28]. Culture characteristics were determined using different ISP media as described by Shirling and Gottlieb [29] and similar to the approach described by Wink *et al.* [30]. Colony colour was determined using Baumann's Farbatlas 1 (Paul Baumann/Aue). Carbohydrate utilization was determined using ISP9 (carbon utilization medium), supplemented with 1 % sole carbon source, as positive control served ISP9 supplemented with 1 % glucose. Melanoid pigment production was examined on peptone–yeast extract iron agar (ISP6), tyrosine agar (ISP7) and a synthetic medium from Suter [31] with and without tyrosine (1 g l^−1^). Sodium chloride tolerance was tested on ISP2 with concentrations from 1–15% and pH tolerance (range, pH 4–10) was tested in ISP2 broth using the buffer system described by Xu *et al.* [32]. Gram-staining was performed according to the standard method [33]. Decomposition of purines, tyrosine and organic acids was tested using the method described by Gordon *et al.* [34]. Antibiotic susceptibility was tested on ISP2 agar [35]. Antibiotics were purchased from Bio-Rad, bioMérieux, Difco, BD and BBL. Anaerobic and microaerophilic growth was tested by cultivating the strains at 28 °C in chambers with anaerobic or microaerophilic atmosphere generated by GENbox anaerob or GENbox microaer (bioMérieux cat. nos. 96124 and 96125).

For chemotaxonomic analyses, freeze-dried cells were obtained from culture grown in liquid ISP2 medium for 3 days at 28 °C on a rotary shaker at 180 r.p.m. The diagnostic diamino acid of the cell wall was determined in whole-cell hydrolysates by paper chromatography according to Hasegawa [36]. Whole-cell sugars were examined according to Schumann [37]. The occurrence of mycolic acids was detected by TLC as described by Minnikin *et al.* [38]. Respiratory quinones of the strains were extracted, purified and identified as described by Collins *et al.* [39] and Wink *et al.* [40]. Polar lipids were extracted by the method of Minnikin *et al.* [41] and identified by two-dimensional thin-layer chromatography as described by Collins *et al.* [42]. Extraction and analysis of fatty acids was done by the DSMZ Identification service by described standard methods [43].

**Fig. 2.**
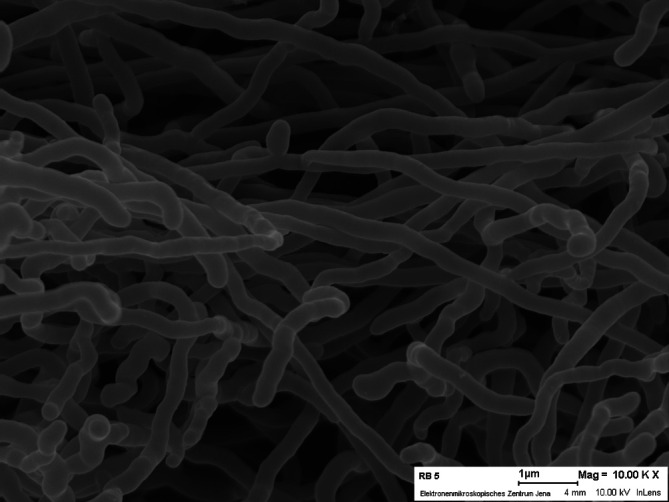
Scanning electron micrograph of strain RB5^T^ (a) cultivated at 28 °C on ISP2 agar for 14 days.

Strain RB5^T^ showed good growth on all tested media and differentiating phenotypic characteristics are shown in Table 1 (Fig. S2, Table S3). White aerial mycelium was well developed on ISP2 and ISP6, poorly developed on ISP5, and no aerial mycelium occurred on ISP1, ISP3, ISP4, ISP7 and Suter medium. Substrate mycelium was well developed on all media with an average hyphal diameter of 0.7–1.1 µm. The substrate mycelium was light green (ISP1–ISP5, ISP7), dark green (ISP6) to beige (Suter Medium). Sporulation was not observed on the tested media (Fig. 2). Phenotypic characterization revealed that strain RB5^T^ grew at pH 6–8 (optimum, pH 7.0) and at 0–1 % (w/v) NaCl (optimum, 0–1 %). Strain RB5^T^ grew well at 20–37 °C and weak growth was observed at 15 and 45 °C. Optimal growth temperature was 28 °C. Decomposition of malate, pyruvate and hypoxanthine was detected. The strain showed only resistance against penicillin G (Table S4).

**Table 1.** Physiological properties that separate the strain RB5^T^ from the type strain of phylogenetically closest related *Streptomyces* species *Streptomyces polyrhachis* DSM 42102^T^ Strains: 1, RB5^T^; 2, *Streptomyces polyrhachis* DSM 42102^T^. Utilization tests are analysed as follows: ++, grows better than positive control, +, grows like positive control (basal medium with glucose); (+), better than negative control but not like positive control; −, not better than negative control (basal medium with water). All strains were positive for utilization of d-glucose. Decomposition of purines, tyrosine and organic acids: −, no decomposition; (+), weak decomposition; +, decomposition; ++, very good decomposition

Characteristics	1	2
Growth at pH	6–8	6–9
Optimal pH	7	7
Temperature growth range (°C)	15–45	15–37
Temperature (°C) for optimal growth	28	28
Anaerobic growth	−	−
Microaerophilic growth	+	+
Growth at NaCl concentration (% w/v)	0–1	0–3
Optimal NaCl concentration (% w/v)	0–1	1
Main menaquinones	MK-9(H_6_), MK-9(H_8_)	MK-9(H_6_), MK-9(H_8_)
Main fatty acids	anteiso-C_15 : 0_, iso-C_16 : 0_	iso-C_16:0_, anteiso-C_15 : 0_
G+C content (mol%)	72.0	74.3
Utilization of sole carbon sources:		
Sucrose	−	+
l-Arabinose	+	−
d-Xylose	+	−
Inositol	−	−
d-Mannitol	−	−
d-Fructose	−	+
l-Rhamnose	+	−
Raffinose	−	−
Cellulose	−	−
Decomposition of purines, tyrosine and organic acids:		
Citrate	−	(+)
Lactate	−	−
Acetate	−	−
Propionate	−	(+)
Malate	++	+
Pyruvate	+	+
Tyrosine	−	−
Adenine	−	+
Hypoxanthine	+	+
Xanthine	−	−

Whole-cell hydrolysates of strain RB5^T^ contained ll-diaminopimelic acid; diagnostic sugars and mycolic acids in whole-cell hydrolysates were not detected. The menaquinone profile of RB5^T^ consisted of MK-9(H_6_), MK-9(H_8_) and traces MK-9(H_4_) in a ratio of 49 : 25 : 5 whereas *S. polyrhachis* DSM42102 exhibited MK-9(H_6_), and MK-9(H_8_) and MK-9(H_4_) in a ratio of 55 : 30 : 15. The polar lipid profile of RB5^T^ consisted of the major compounds diphosphatidylglycerol, phosphatidylethanolamine, phosphatidylinositol, phosphatidylserine, phosphatidylinositol-mannoside, an unidentified glycolipid, an unidentified aminophospholipid and six unidentified phospholipids (PL1–PL6), an aminolipid (AL1) and an unidentified lipid (L1) (Fig. S3).

The fatty acid profile of strain RB5^T^ (Table S4) was composed of the major fatty acids anteiso-C_15 : 0_ (44.8 %), iso-C_16 : 0_ (19.4 %), iso-C_14 : 0_ (13.0 %) and C_15 : 0_ (6.6 %). In contrast, the fatty acid profiles of the closest relative *S. polyrhachis* DSM 42102^T^ contain lower amounts of anteiso-C_15 : 0_ (21.9 %) and iso-C_14 : 0_ (11.2 %). Higher amounts of iso-C_16 : 0_ (38.9 %), anteiso-C_17 : 0_ (8.4 %) and iso-C_16 : 1_ *ω6*c (7.1 %) were detected in *S. polyrhachis* DSM 42102^T^ (Table S5).

The analyses of fatty acid profiles clearly differentiated the new isolate from its closest relative. Subsequent analyses of the culture characteristics of both strains supported this assumption.

Morphological, physiological, genetic and chemotaxonomic data support the delineation of RB5^T^ as representing a novel species of the genus *Streptomyces.*

## Description of *Streptomyces smaragdinus* sp. nov.

*Streptomyces smaragdinus* (sma.rag.di'nus. L. masc. adj. *smaragdinus*, smaragd-green-coloured, referring to the smaragd-green-coloured substrate mycelium on ISP2 and ISP6 media).

Aerobic, Gram-stain-positive actinobacterium that forms branched, vegetative mycelium. Good growth occurs on ISP1–ISP7 and Suter medium with an average hyphal diameter of 0.7–1.1 µm. Aerial mycelium is forms poorly on ISP2, ISP5 and ISP6 within 12–14 days. A dark green colour is observed on ISP6. Spore formation is not detectable on ISP1–ISP7 or Suter medium.

The pH range for growth is pH 6–8 (optimum, pH 7.0). The maximum concentration of NaCl for growth is 1 % (w/v) supplemented on ISP2. Growth occurs between 15–45 °C on ISP2 agar with an optimum at 28 °C. The strain grows with glucose, l-arabinose, d-xylose and l-rhamnose as a sole carbon source, but not with sucrose, inositol, mannitol, fructose, raffinose or cellulose. Malate, pyruvate and hypoxanthine are decomposed. The strain is resistant to penicillin G.

The diagnostic amino acid of the peptidoglycan is ll-diaminopimelic acid. Mycolic acids and diagnostic sugars in whole-cell hydrolysates are not detected. The major polar lipids are diphosphatidylglycerol, phosphatidylethanolamine, phosphatidylinositol, phosphatidylserine, phosphatidylinositol-mannoside, an unidentified glycolipid, an unidentified aminophospholipid, six unidentified phospholipids, an unidentified aminolipid and the unidentified lipid L1. The major fatty acids are anteiso-C_15 : 0_ (12-methyltetradecanoic acid), iso-C_16 : 0_ (iso-hexadecanoic acid), iso-C_14 : 0_ (isomyristic acid) and C_15 : 0_ (pentadecanoic acid). The predominant menaquinones are MK-9(H_6_) and MK-9(H_8_). The DNA G+C content of the type strain is 72 mol%.

The type strain, RB5^T^ (=VKM Ac-2839^T^=NRRL B-65539^T^), was isolated from the gut of the termite *Macrotermes natalensis* (major worker). The GenBank/EMBL accession number for the partial 16S rRNA gene sequence of strain RB5^T^ is KY558696.2. This Whole Genome Shotgun project has been deposited at DDBJ/ENA/GenBank under the accession WEGJ00000000 (Bio Project PRJNA545686,; Bio Sample SAMN11898449). The version described in this paper is WEGJ01000000.

## Supplementary Data

Supplementary material 1
