# Supplementary material for: Streptomyces smaragdinus sp. nov., isolated from the gut of the fungus growing-termite Macrotermes natalensis
Source: Int J Syst Evol Microbiol. 2020 Sep 24;70(11):5806–11. doi: 10.1099/ijsem.0.004478 (PMC7723250; doi:10.1099/ijsem.0.004478)
Supplement: Supplementary material 1 [file ijsem-70-5806-s001.pdf]

Streptomyces smaragdinus sp. nov., isolated from the gut of the fungus growing-termite *Macrotermes natalensis*

Jan W. Schwitalla,[1#] René Benndorf,[1#] Karin Martin,[1*] John Vollmers,[2] Anne-Kristin Kaster,[2] Z. Wilhelm de Beer,[3] Michael Poulsen,[4] and Christine Beemelmanns[1*]

[1]Leibniz Institute for Natural Product Research and Infection Biology e. V., Hans-Knöll-Institute, Beutenbergstraße 11a, 07745 Jena, Germany

[2]Institute for Biological Interfaces (IBG 5), Karlsruhe Institute of Technology, Hermann-von-Helmholtz-Platz 1, 76344 Eggenstein-Leopoldshafen, Germany

[3]Department of Microbiology and Plant Pathology, Forestry and Agriculture Biotechnology Institute, University of Pretoria, 0028 Hatfield, South Africa

[4] University of Copenhagen, Department of Biology, Section for Ecology and Evolution, Universitetsparken 15, 2100 Copenhagen East, Denmark

Correspondence

Christine Beemelmanns

E-mail: christine.beemelmanns@leibniz-hki.de

Karin Martin

E-mail: karin.martin@leibniz-hki.de

#Authors contributed equally to the manuscripts

Subject category: New Taxa; **Subsection:** Gram-positive bacteria

Keywords: *Streptomyces*, *Macrotermes natalensis*, termite gut

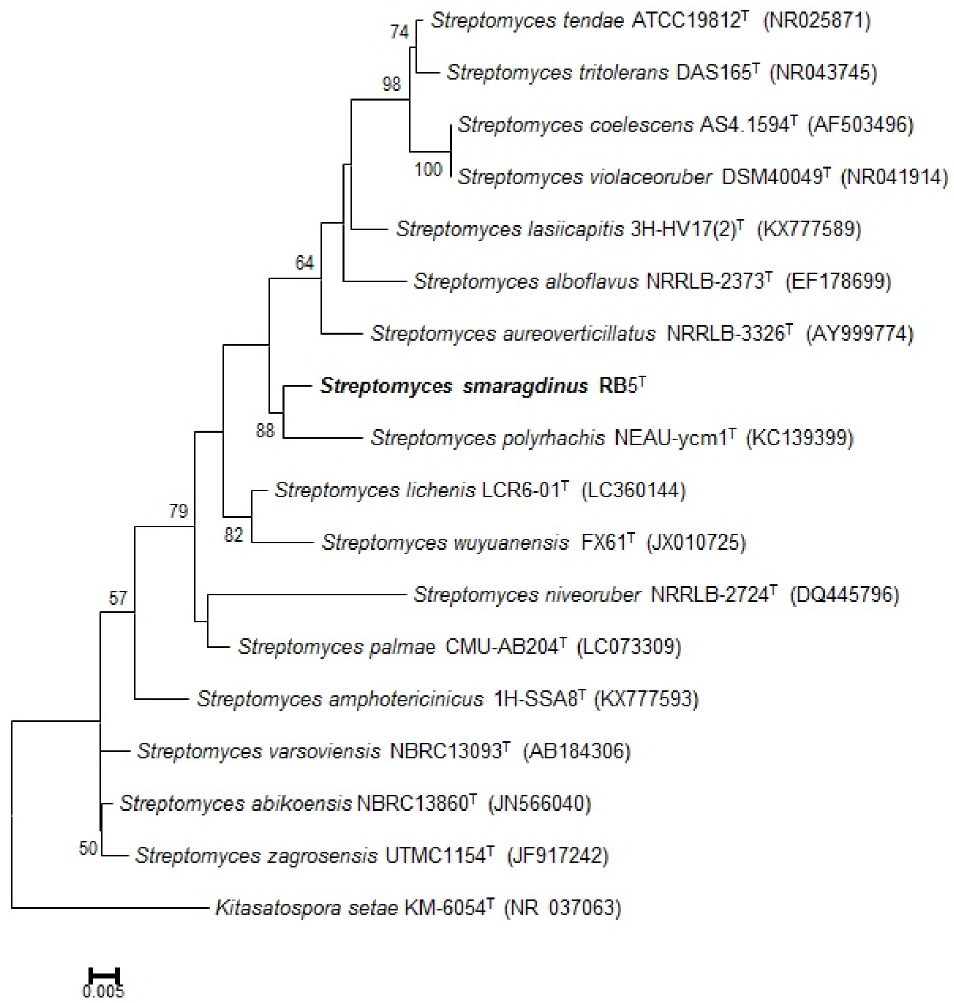

Figure S1. Maximum-likelihood phylogenetic tree showing the phylogenetic relationship of strain RB5^{T} and other closely related species based on 16S rRNA gene sequences. *Kitasatospora setae* KM-6054^{T} was used to root the tree. Only bootstrap values above 50% (1000 pseudoreplications) are shown. Bar length corresponds to 0.005 substitutions per nucleotide position.

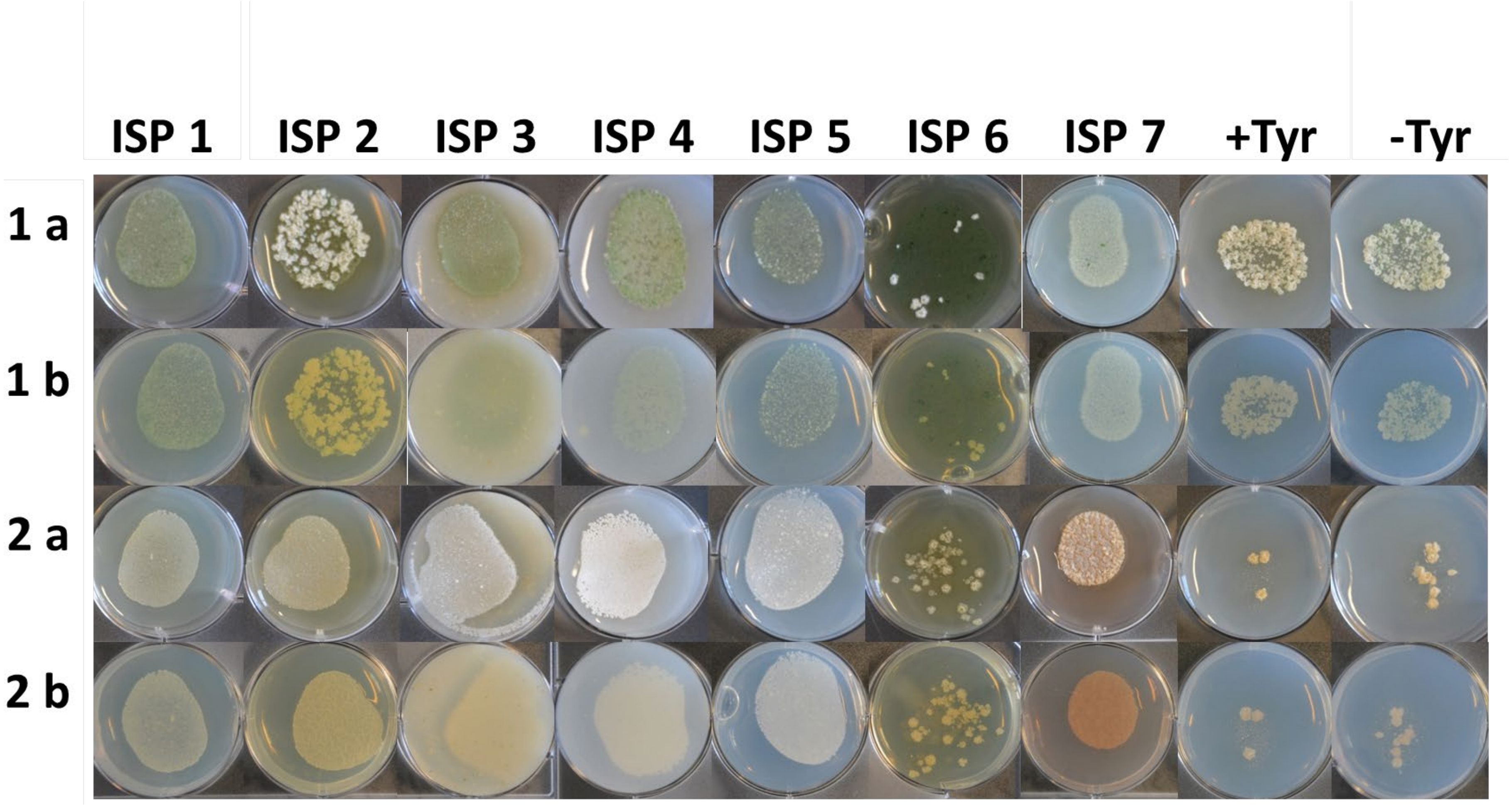

Figure S1. Morphology of strains grown on different ISP-media and Suter-Medium (with: +Tyr [1 g/L] and without tyrosine: -Tyr) for 12 days at 28 °C. Strains: RB5^T (1a: above, 1b: reverse); *Streptomyces polyrhachis* DSM42102^T (2a: above, 2b: reverse).

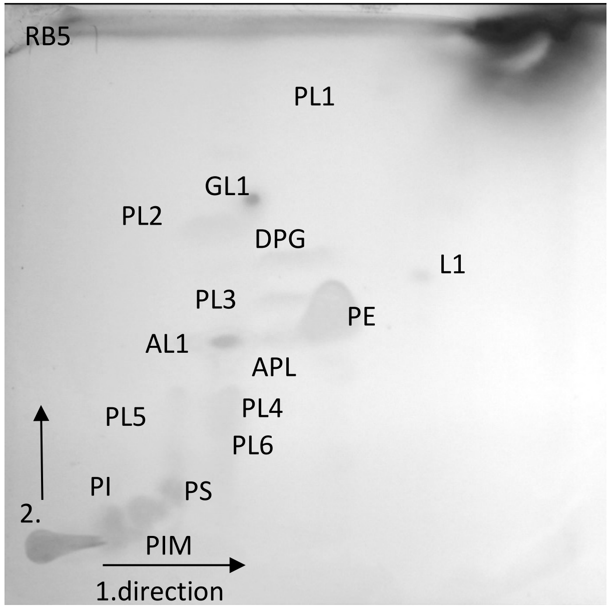

Fig S3. Polar lipid profile of strain RB5^T after two-dimensional TLC and detection with molybdatophosphoric acid. The solvent system consisted of chloroform-methanol-water (65:25:4, by vol.) for the first development and of chloroform-methanol-glacial acetic acid-water (80:12:15:4, by vol.) for second development. DPG, diphosphatidyglycerol; PE, phosphatidylethanolamine; PI, phosphatidylinositol; PIM, phosphatidylinositol mannoside; PS, phosphatidylserine; AL1, unknown aminolipid; APL, aminophospholipid; PL1-PL6, unidentified phospholipids; GL1 unidentified glycolipid; L1, unidentified lipid

Table S1. Calculated sequence similarities of strain RB5^T and *Streptomyces* strains

**Strain (Accession number)**	**Similarity [%]**
Streptomyces polyrhachis NEAU-ycm1^T (KC139399)	98.87
Streptomyces aureoverticillatus NRRL B-3326^T (AY999774)	98.52
Streptomyces alfalfae XY25^T (KR080524.1)	98.37
Streptomyces variegatus LMG 20315^T (AJ781371)	98.37
Streptomyces tendae ATCC 19812^T (D63873)	98.27
Streptomyces pluripotens MUSC 135^T (KF195922)	98.24
Streptomyces marokkonensis Ap1^T (AJ965470)	98.23
Streptomyces tritolerans DAS 165^T (DQ345779.2)	98.20
Streptomyces coelescens AS 4.1594^T (AF503496)	98.20
Streptomyces violaceoruber CSSB 1016^T (AF503492)	98.20
Streptomyces humiferus DSM 43030^T (AF503491)	98.20
Streptomyces violaceolatus ATCC 19847^T (AF503497)	98.19
Streptomyces intermedius NBRC 13049^T (AB184277)	98.16
Streptomyces gougerotii NBRC 3198^T (AB184742)	98.16

[b] Sequence similarities were calculated using the method recommended by Meier-Kolthoff (1)

1. Meier-Kolthoff JP, Goker M, Sproer C, Klenk HP. When should a DDH experiment be mandatory in microbial taxonomy? Arch Microbiol. 2013b;195(6):413-8.

Table S2. Summary of *Streptomyces* genome sequenced in this work, including strain ID, genus, total genome size (in mega base pairs), GC content in %, number of assembled contigs (contiguous sequences)**; for more details, see**

https://www.ncbi.nlm.nih.gov/nuccore/1774106366

**Strain ID**	**RB5**
Genus	Streptomyces
Total size [Mb]	~7.9
GC content [%]	72.00
Number of contigs	107
N50 [bp]	161.565
L50	13
Total CDS	7136
Estimated completeness [%]	100.00
Estimated contamination	1.32
Assembly Date :: 2017<br>Assembly Method :: SPAdes v. 3.10.1<br>Genome Representation :: Full<br>Expected Final Version :: Yes<br>Genome Coverage :: 100.0x<br>Sequencing Technology :: Illumina MiSeq<br>Annotation Provider :: NCBI RefSeq<br>Annotation Date :: 11/08/2019 04:06:55<br>Annotation Pipeline : NCBI Prokaryotic Genome Annotation Pipeline (PGAP)<br>Annotation Method :: Best-placed reference protein set; GeneMarkS-2+<br>Annotation Software revision :: 4.10<br>Features Annotate: Gene; CDS; rRNA; tRNA; ncRNA; repeat_region<br>Genes (total) :: 7,155<br>CDSs (total) :: 7,074<br>Genes (coding) :: 6,671<br>CDSs (with protein) :: 6,671<br>Genes (RNA) :: 81<br>rRNAs :: 5, 1, 2 (5S, 16S, 23S)<br>complete rRNAs :: 3, 1, 1 (5S, 16S, 23S)<br>partial rRNAs :: 2, 1 (5S, 23S)<br>tRNAs :: 70<br>ncRNAs :: 3<br>Pseudo Genes (total) :: 403<br>CDSs (without protein) :: 403<br>Pseudo Genes (ambiguous residues) :: 0 of 403<br>Pseudo Genes (frameshifted) :: 55 of 403<br>Pseudo Genes (incomplete) :: 357 of 403<br>Pseudo Genes (internal stop) :: 13 of 403<br>Pseudo Genes (multiple problems) :: 22 of 403<br>CRISPR Arrays :: 2	

Table S3. Growth characteristics of strain RB5^T and the type strain of closest related *Streptomyces* species after 12 to 14 days of incubation at 28 °C. Strains: 1. RB5^T; 2. *S. polyrhachis* DSM42102^T; all data was acquired in this study. Morphological feature: G: growth, AM: aerial mycelium, SM: substrate mycelium, SP: soluble pigment, Colour coding (N.) corresponding to Baumann´s Farbatlas 1 in parentheses.

[illegible]	[illegible]	1	2
ISP1	G	Good	Good
	AM	None	None
	SM	Light green , (No.577-580 NE,1)	Cream white (No.4)
	SP	None	None
ISP2	G	Good	Good
	AM	White	None
	SM	Light green, white (No.577-580 NE)	Cream white (No.4)
	SP	None	None
ISP3	G	Good	Good
	AM	None	Very poor, white
	SM	Light green, white (No.577-580 NE)	Cream white (No.4)
	SP	None	None
ISP4	G	Good	Good
	AM	None	None
	SM	Light green, (No.577-580 NE)	White
	SP	None	None
ISP5	G	Good	Good
	AM	Poor on margin, white	Very poor, white
	SM	Light green (No.577-580 NE)	White
	SP	None	None
ISP6	G	Good	Good
	AM	White	None
	SM	Dark green (No.590-593)	Yellowish
	SP	None	None
ISP7	G	Good	Weak
	AM	None	Very poor, white
	SM	Light green , (No.577-580 NE)	Orange (No.4)
	SP	None	None
medium with	G	Good	Good
	AM	None	None
	SM	Beige	Cream white (No.4)
	SP	None	Reddish brown (No.173)
medium without	G	Good	Good
	AM	None	None
	SM	Beige	Cream white (No.4)
	SP	None	None

Table S4. Antibiotic susceptibility test of strains RB5^T and *S. polyrhachis* DSM 42102^T after 12 days of incubation at 28 °C. The diameter of the inhibition zone is given in mm.

Antibiotic	Conc.	1	2
Amikacin	30 µg	50	24
Ampicillin	10 µg	24	0
Azlocillin	75 mg	10	0
Bacitracin	130 µg	40	30
Cephalothin	30 µg	11	0
Imipenem	10 µg	60	21
Mezlocillin	75 µg	24	0
Penicillin G	10 units	0	0
Piperacillin	100 µg	20	0
Polymyxin B	30 units	12	0
Vancomycin	30 µg	32	23
Chloramphenicol	30 µg	37	20
Chlortetracycline	30 µg	24	26
Doxycycline	30 µg	28	24
Erythromycin	15 µg	24	40
Gentamicin	10 µg	19	9
Kanamycin	30 µg	46	20
Lincomycin	2 µg	8	0
Oxytetracycline	30 µg	17	15
Rifampin	5 µg	36	35
Streptomycin	10 µg	37	15
Tetracycline	30 µg	16	40
Ciprofloxacin	5 µg	24	30
Norfloxacin	10 µg	10	12
Novobiocin	5 µg	10	25
Nalidixic acid	30 µg	10	0

Table S5. Cellular fatty acid compositions of strain RB5^T and close related type strains of *Streptomyces polyrhachis.* Strains: 1. RB5^T; 2. *S. polyrhachis* DSM 42102^T. Amounts of fatty acids below 1.0% were not listed or marked with tr = traces. All data were acquired in this study.

**Fatty acids**	**1**	**2**
Saturated fatty acids		
14:0	tr	tr
15:0	6.6	tr
16:0	tr	2.7
Unsaturated fatty acids		
16:1 cis 9	tr	tr
iso-16:1 *ω6cis*	2.1	7.1
anteiso-17:1 *ω7cis*	tr	tr
Sum 17:1 ISO *ω7cis* /17:1 ISO *ω9cis*	-	-
Branched fatty acids		
iso-14:0	13.0	11.2
iso-15:0	4.1	4.1
iso-16:0	19.4	38.9
iso-17:0	tr	tr
anteiso-15:0	44.8	21.9
anteiso-17:0	3.9	8.4
